# A mechanistic approach to prove the efficacy of combination therapy against New Delhi metallo-β-lactamases producing bacterial strain: a molecular and biochemical approach

**DOI:** 10.1186/s40001-020-00418-1

**Published:** 2020-06-03

**Authors:** Lubna Maryam, Abid Ali, Shamsi Khalid, Asad U. Khan

**Affiliations:** Medical Microbiology and Molecular Biology Laboratory, Interdisciplinary Biotechnology Unit, Aligarh Muslim University, Aligarh, Uttar Pradesh 202 002 India

**Keywords:** Streptomycin, Amikacin, Ciprofloxacin, Synergistic effect, Antibiotic resistance, Fractional inhibitory concentration

## Abstract

**Background:**

NDM-1 is a novel broad-spectrum metallo-β-lactamase with the capability to grant resistance to almost all β-lactam antibiotics. Its widespread dissemination made treatment options a major challenge to combat, causing threat to public health worldwide. Due to antibiotic resistance problems, development of effective therapeutics for infections caused by NDM-1 producing strains is urgently required. Since combination therapies are proved to be effective in many cases, this study was initiated to put forward novel effective antibiotics combinations for fighting infections caused by NDM-1 producing strains.

**Methods:**

Streptomycin and amikacin combination and streptomycin and ciprofloxacin combination were tested by checkerboard assay. NDM-1 protein/enzyme was then expressed and purified to carry out enzyme kinetics study, CD and fluorescence spectroscopic studies.

**Results:**

Streptomycin and amikacin combination and streptomycin and ciprofloxacin combination showed synergistic effect towards NDM-1 producing bacterial strains as shown by FICI results. NDM-1 producing bacterial cells were expressed and purified to obtain protein as the source of enzyme. When NDM-1 enzyme was treated with streptomycin along with amikacin, the efficiency of enzyme was decreased by 49.37% and when the enzyme was treated with streptomycin along with ciprofloxacin, the efficiency of enzyme was decreased by 29.66% as revealed by enzyme kinetic studies. Due to binding of streptomycin and amikacin in combination and streptomycin and ciprofloxacin in combination, conformational changes in the secondary structure of NDM-1 enzyme were observed by CD spectroscopic studies. Antibiotics streptomycin and ciprofloxacin bind with NDM-1 through exothermic processes, whereas amikacin binds through an endothermic process. All three antibiotics bind spontaneously with an association constant of the order of 10^4^ M^−1^ as revealed by fluorescence spectroscopic studies.

**Conclusions:**

The therapeutic combination of streptomycin with amikacin and ciprofloxacin plays an important role in inhibiting NDM-1 producing bacterial strains. Therefore, these combinations can be used as effective future therapeutic candidates against NDM-1 producing bacterial cells.

## Background

With the rise in antimicrobial resistance, the reserves of potent antibiotics are depleted raising the occurrence of untreatable infection. With the emergence of β-lactamases producing bacteria, advances in the development of newer antibiotic agents have failed to keep its pace. Carbapenemases are highest resistance producers among the β-lactamases. Several bacteria such as *Escherichia coli*, *Klebsiella pneumoniae*, etc., belonging to Gram-negative category produce a β-lactamase enzyme called as NDM-1 (New Delhi metallo-β-lactamase 1) which is responsible for resistance to a broad range of antibiotics belonging to the β-lactam group [1]. NDM-1 gene encodes a β-lactamase enzyme called as carbapenemases. They have versatile capacity to hydrolyze almost all β-lactams (penicillins, cephalosporins, carbapenems, monobactams) and classic new β-lactamase inhibitors such as tazobactam, sulbactam, avibactam, etc., rendering them ineffective for treating serious bacterial infections [2]. NDM-1 belongs to metallo-β-lactamases that have divalent zinc cation and water molecule regulating its active site [3]. Bacteria producing NDM-1 invades blood, urinary tract, wounds and lungs causing septicaemia. These bacteria also cause urinary tract infection, soft tissue infection, peritonitis, gastrointestinal problems, pulmonary infections, etc. [4]. The worldwide emergence and dissemination of NDM-1 producing bacteria and its variants and the problem of resistance to antibiotics associated with it has raised a serious public health concern [5]. It is therefore, the need of the hour to develop suitable antimicrobials against NDM-1 producing bacterial strains.

Aminoglycoside (streptomycin, amikacin, etc.) class of antibiotics is used against the infections caused by Gram-negative bacteria either singly or in combination [6]. These antibiotics are administered either intravenously or intramuscularly and act by inhibiting protein synthesis. Amikacin is reported to be least susceptible to degradation by the enzymes produced by bacteria [6]. However, it is reported that there is an increase in aminoglycoside resistance lately [7]. Several bacteria produce plasmid-encoded and transposable elements associated aminoglycoside-modifying enzymes that play role in catalyzing covalent modification of aminoglycoside leading to failure in the uptake of antibiotics and occurrence of high-level resistance [8]. Aminoglycosides are also reported to be commonly used in combination with β-lactams and other agents to make use of synergism between them in several Gram-negative infections [9].

Quinolone group of antibiotics is a large group of bactericides used in the treatment of various bacterial infections like urinary tract infection, respiratory tract infection, etc., caused by both Gram-positive and negative bacteria in humans. Almost all quinolone antibiotics in use contain fluorine atom in their molecular structure and are therefore referred to as fluoroquinolone. These antibiotics have fine tissue penetration, outstanding oral bioavailability, good tolerability and safety profiles [10]. One of the most widely used antibiotic ciprofloxacin belongs to second-generation quinolones category of antibiotics with increased systemic and Gram-negative activity [10, 11]. These antibiotics work by preventing bacterial DNA synthesis process [12]. However, due to the broad use of fluoroquinolones, the emergence of resistance is observed mainly because of occurrence of mutations in the gene encoding the target sites of drug [12]. Combination therapies of ciprofloxacin with other antibiotics like rifampicin have been proved to be effective for treatment [13].

To combat resistance against antibiotics while treating infections caused by multi-drug-resistant strains, combination therapy using two agents having a different mechanism of antibacterial action is recommended [14]. Using antibiotics in combination against multi-drug-resistant strains is reported to be a promising strategy in treatment options [15–18]. With the rise in infections, caused by multi-drug-resistant Gram-negative bacteria, combination therapy is employed in many healthcare facilities for severely ill patients with neutropenia, *Pseudomonas aeruginosa* infections, ventilator-associated pneumonia, etc. [19]. Since single antibiotic has become ineffective for treating infections caused by NDM-1 producing bacterial strains, combination therapy with two antibiotics at a time should be checked for its therapeutic action. It is reported that aminoglycosides can be opted for critically ill patients with serious infections along with β-lactams or fluoroquinolones [20]. Also, combination therapy of ciprofloxacin with gentamicin (an aminoglycoside) has been reported to be effective in treatment options [21].

However, using two aminoglycosides for treating infections caused by Gram-negative multi-drug-resistant strains have not been reported yet, therefore this study was initiated to check the effect of combination therapy using two aminoglycosides (streptomycin and amikacin) as well as combination of aminoglycoside and quinolone (ciprofloxacin) against NDM-1 producing bacterial strains along with the mechanism behind their action on this target.

## Methods

### Vector and strains used

Minimum inhibitory concentration (MIC) and fractional inhibitory concentration index (FICI) were determined using *Klebsiella pneumoniae* AK-66 strain having NDM-1 gene on its plasmid (Genebank accession no.: KX231906.1). For cloning of NDM-1 gene, NDM-1 gene was extracted from *Klebsiella pneumoniae* strain having NDM-1 gene, pQE-2 was used as vector and *E. coli* (λDE3)BL21 cells were used as competent cells.

### Antibiotics/chemicals used

Streptomycin and amikacin were purchased from Himedia (Mumbai, India), ciprofloxacin was purchased from Sigma-Aldrich. Isopropyl-β-thiogalactopyranoside (IPTG) used as an inducer for expression of the NDM-1 protein, purchased from Roche (Basel, Switzerland). Nitrocefin used as a substrate for getting NDM-1 β-lactamase hydrolytic profile was purchased from Calbiochem (USA). High purity chemicals, antibiotics and buffer were used throughout the study. All the experiments were carried out using double distilled water.

### NDM-1 protein expression and purification

For expression and purification of NDM-1 protein/enzyme previously cloned *E. coli* BL21 cells harboring NDM-1 gene were used [22]. Primary culture of cells was grown in 1-l culture of Luria–Bertani broth supplemented with 100 µg/ml of ampicillin at 37 °C and 180 rpm, till OD (optical density/absorbance) of 0.4 to 0.6 was reached at 600 nm wavelength. Once the cells reach log phase/exponential phase at 0.4 to 0.6 OD, 0.5 mM IPTG was used for 3 h at 37 °C as an inducer to express the NDM-1 protein [22]. Cells were then centrifuged to collect cell the pellet. NDM-1 protein was then purified using affinity chromatography via protocol described earlier [23]. After protein purification dialysis was carried out at 4 °C in HEPES buffer (50 mM, pH 7.0) along with NaCl (250 mM) and ZnCl_2_ (100 µM) to obtain pure protein. Using molar extinction coefficient of 27,880 M^−1^cm^−1^, the concentration of the purified protein was determined at 280 nm using UV spectrophotometer. Further by using SDS-PAGE purity of the purified NDM-1 protein was checked [24].

### MIC test

Using microdilution method and guidelines laid by Clinical Laboratory Standards Institute [25], the MIC (minimum inhibitory concentration) of streptomycin, amikacin and ciprofloxacin were determined for *Klebsiella pneumoniae* (AK-66) strain harboring NDM-1 gene on its plasmid. The lowest concentration of antibiotics streptomycin, amikacin and ciprofloxacin that inhibited the visible growth of NDM-1 carrying bacteria completely was reported as the MIC of that antibiotic.

### 2D-checkerboard microdilution assay

2D-checkerboard microdilution assays were carried out using 96-well microtiter plates to identify the kind of interaction occurring between streptomycin and amikacin with NDM-1 gene harboring *Klebsiella pneumoniae* (AK-66) strain and between streptomycin and ciprofloxacin with *Klebsiella pneumoniae* (AK-66) strain having NDM-1 marker, respectively. Streptomycin and amikacin, and streptomycin and ciprofloxacin were serially diluted in concentrations less than, equal to and above their MICs. To know the effect of streptomycin and amikacin combination and streptomycin and ciprofloxacin combination towards NDM-1-harboring bacterial strain, FIC (fractional inhibitory concentration) and FICI (FIC index) using the following Eqs. 1 and 2 [26]:

1 $$\begin{aligned} {\text{FIC of streptomycin}} & = \, \left( {\text{MIC of streptomycin in combination}} \right)/\left( {\text{MIC of streptomycin}} \right) \\ {\text{FIC of amikacin}} & = \, \left( {\text{MIC of amikacin in combination}} \right)/\left( {\text{MIC of amikacin}} \right) \\ {\text{FICI }} & = {\text{ FIC of streptomycin }} + {\text{ FIC of amikacin}} \\ \end{aligned}$$

2 $$\begin{aligned} {\text{FIC of streptomycin }} & = \, \left( {\text{MIC of streptomycin in combination}} \right)/\left( {\text{MIC of streptomycin}} \right) \\ {\text{FIC of ciprofloxacin }} & = \, \left( {\text{MIC of ciprofloxacin in combination}} \right)/\left( {\text{MIC of ciprofloxacin}} \right) \\ {\text{FICI }} & = {\text{ FIC of streptomycin }} + {\text{ FIC of ciprofloxacin}} \\ \end{aligned}$$

### Steady-state NDM-1 enzyme kinetics

Steady-state NDM-1 enzyme kinetic studies were carried out to know the effect of streptomycin in combination with amikacin binding and streptomycin in combination with ciprofloxacin binding on the catalytic activity and efficiency of the NDM-1 enzyme. For this, a cephalosporin substrate nitrocefin which gives a characteristic β-lactamase (NMD-1 in this study) hydrolytic profile, was used to monitor the activities of the NDM-1 enzyme in the absence and presence of respective antibiotics in single and combination. The activities of 5 nM of NDM-1 enzyme towards nitrocefin in the absence of any antibiotic, in the presence of 5 nM of streptomycin, in the presence of 5 nM of amikacin and in the presence of 5 nM each of streptomycin and amikacin taken together, were measured by varying the concentration of nitrocefin from 0 to 700 µM in 50 mM sodium phosphate buffer of pH 7.4 at 298 K. Similarly, another enzyme kinetics profile was carried out by measuring the activities of 5 nM of NDM-1 enzyme towards nitrocefin in the absence of any antibiotic, in the presence of 5 nM of streptomycin, in the presence of 5 nM of ciprofloxacin and in the presence of 5 nM each of streptomycin and ciprofloxacin in combination by varying the concentration of nitrocefin from 0 to 700 µM in 50 mM sodium phosphate buffer (pH 7.4) at 298 K. For the dilution of NMD-1 enzyme and to prevent its denaturation, 20 µg/ml of BSA was added since; at this concentration of BSA, the activity of the enzyme is not influenced [27]. The experiments were carried out on UV-1800 spectrophotometer purchased from Shimadzu International co. Ltd, Kyoto, Japan. Hydrolysis of nitrocefin by the NDM-1 enzyme in the absence and presence of antibiotics in single and in combination was measured at 486 nm wavelength for 70 s using molar extinction coefficient as 15,000 M^−1^cm^−1^. Kinetic parameters like Michaelis–Menten constant (*K*_m_), catalytic activity (*K*_cat_) and catalytic efficiency (*K*_cat_/*K*_m_) were deduced using the following Eqs. 3 and 4:

3 $$v = \frac{{V_{ \text{max} } \left[ S \right]}}{{K_{\text{m}} + \left[ S \right]}},$$

4 $$K_{\text{cat}} = \frac{{V_{ \text{max} } }}{\left[ E \right]}.$$

### Far-UV CD spectroscopy

To monitor the effect on the conformation of the NDM-1 protein upon binding of streptomycin, amikacin, ciprofloxacin, streptomycin in combination with amikacin and streptomycin in combination with ciprofloxacin, Circular Dichroism (CD) spectroscopy was carried out in far-UV range. The study was done at 298 K by setting scan speed at 100 nm/min and response time of 1 s, using 0.1 cm pathlength cuvette on spectropolarimeter (J-815 Jasco from International Co. Ltd, Tokyo, Japan) having Peltier-type temperature controller (PTC-423S/15). The experiment was started after calibrating the instrument using (+)-10-camphor sulfonic acid. Far-UV CD spectra in between 200 and 250 nm wavelengths were taken of 5 µM of NDM-1 protein in the absence of any antibiotic, in the presence of 5 µM of streptomycin, in the presence of 5 µM of amikacin and in the presence streptomycin in combination with amikacin in 5 µM concentration each. In the same way, another CD spectra profile were obtained by taking 5 µM of NDM-1 protein in the absence of any antibiotic, NDM-1 in the presence of 5 µM of streptomycin, NDM-1 in the presence of 5 µM of ciprofloxacin and NDM-1 in the presence of streptomycin in combination with ciprofloxacin in 5 µM concentration each. Correction for appropriate blank was done for each measurement. Observed ellipticity which was obtained from CD measurements was used for the calculation of MRE (mean residual ellipticity) values using the following Eq. 5 [28]:

5 $${\text{MRE}} = \frac{{\left[ \theta \right]{\text{obs}}}}{{10{\text{ncl}}}},$$

where [θ] obs is referred to as observed ellipticity in mdeg, *n* is referred to as the total number of amino acid residues present in the NDM-1 protein, c is the concentration of NDM-1 protein in moles and l is the pathlength in cm. From the calculated MRE values at 222 nm, the α-helical content percentage of NDM-1 protein was calculated in the absence and presence of antibiotics as single and in combination using the following Eq. 6 [29]:

6 $$\% \propto - {\text{helix}} = \left[ {\frac{{\left[ {\text{MRE}} \right]222{\text{nm}} - 2340}}{30300}} \right] * 100$$

### Fluorescence spectroscopy

Understanding the mechanism behind protein–drug interaction is of crucial importance, therefore in order to get an insight into the mechanism of interaction of streptomycin, amikacin and ciprofloxacin with NDM-1 protein at 298, 303 and 308 K, fluorescence spectroscopy was performed on a spectrofluorometer (RF-5301PC, from Shimadzu Corporation, Kyoto, Japan). The instrument was equipped with a cell holder controlled thermostatically and a water bath to maintain required temperature [30]. Fluorescence spectra of 2 μM NMD-1 protein were taken without any antibiotic and with 2 μM of antibiotic (streptomycin, amikacin and ciprofloxacin) added successively till 18 μM concentration is reached in a sample of 450 μl. The spectra were recorded at fast scanning mode between 315 to 450 nm wavelength after exciting the samples at 295 nm by setting both excitation and emission slits at 5 nm. Correction for the inner filter effect was done for each of the fluorescence intensities recorded [31]. Fluorescence intensities at emission maxima were analyzed using the following Stern–Volmer Eq. 7 [32]:

7 $$F{^\circ} /F = 1 + {\text{Ksv}}\left[ {\text{Q}} \right],$$

where Fͦ is the fluorescence intensity of NDM-1 in the absence of any antibiotic and F is the fluorescence intensities of NDM-1 in the presence of varying concentration of antibiotics, *K*_sv_ is the Stern–Volmer constant and [Q] is the concentration of the antibiotic used. In the same way, other binding parameters such as binding constant (Ka) and the number of binding sites available for binding (*n*) were calculated using the following modified Stern–Volmer Eq. 8 [33]:

8 $$\log \frac{F^\circ - F}{F} = \log {\text{Ka}} + n\log \left[ Q \right].$$

Further to obtain the thermodynamic parameters of streptomycin, amikacin and ciprofloxacin interaction with NMD-1 protein, the following Van’t Hoff Eq. 9 and thermodynamic Eq. 10 were used. Using these two equations, enthalpy change (Δ*H*), entropy change (Δ*S*) and Gibbs free energy change (Δ*G*) were calculated:

9 $${\text{lnKa}} = \left( {\frac{\Delta S}{R}} \right) - \left( {\frac{\Delta H}{RT}} \right),$$

10 $$\Delta G = \Delta H - T\Delta S.$$

## Results

**Fig. 1:**
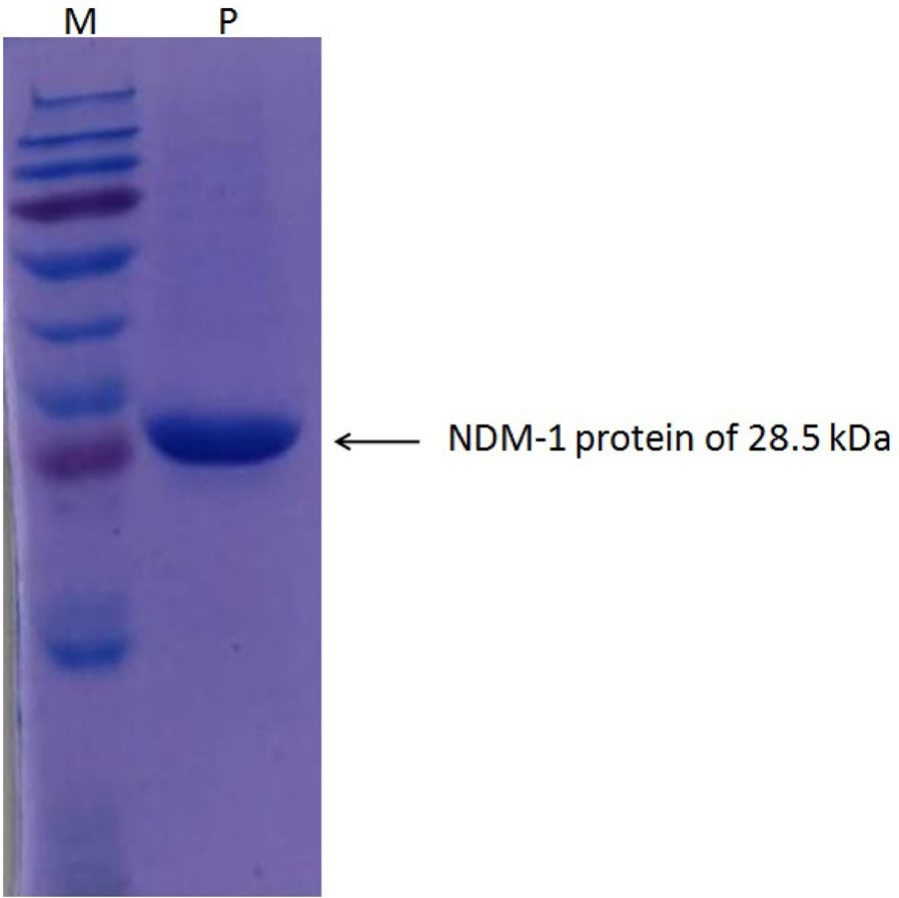
SDS polyacrylamide gel of soluble NDM-1 protein fraction isolated from *E. coli* BL21 cells having the NDM-1 marker. The cells were induced with 0.5 mM IPTG for 3 h at 37 °C. Lane P is the NDM-1 protein of 28.5 kDa in size and lane M is the prestained protein ladder

The isolated NDM-1 protein was found to be of 1.7 mg/ml in concentration with 94% purity. A single band of 28.5 kDa in size was seen on sodium dodecyl sulphate polyacrylamide gel (SDS-PAGE) as shown in Fig. 1.

The minimum inhibitory concentration (MIC) of streptomycin, amikacin and ciprofloxacin for *Klebsiella pneumoniae* AK-66 strain having NDM-1 gene on its plasmid was reported to be 1024 µg/ml, > 1024 µg/ml and 128 µg/ml, respectively, which is in highly resistant range (Table 1).

**Table 1:** MICs of streptomycin, amikacin and ciprofloxacin antibiotics for NDM-1 harboring *K. pneumoniae* AK66 strain

Antibiotics	AK66 *K. pneumoniae* strain harboring NDM-1 gene MIC (µg/ml)
Streptomycin	1024
Amikacin	> 1024
Ciprofloxacin	128

By performing a 2D-checkerboard microdilution assay, the type of interaction occurring between streptomycin and amikacin combination and streptomycin and ciprofloxacin combination with *Klebsiella pneumoniae* AK-66 strain harboring NDM-1 gene on its plasmid was determined. The FICI (fractional inhibitory concentration index) value of streptomycin and amikacin interaction was found to be 0.3125 and of streptomycin and ciprofloxacin interaction was found to be 0.31, respectively (Table 2).

**Table 2:** Possible synergistic combinations obtained by 2D-checkerboard microdilution assay for NDM-1 gene harboring AK66 strain of *K. pneumoniae*

Antibiotic combinations	AK66 strain of *K. pneumoniae* harboring NDM-1 gene FICI values (FICI ≤ 0.5)
Streptomycin + amikacin	0.3125
Streptomycin + ciprofloxacin	0.31

**Fig. 2:**
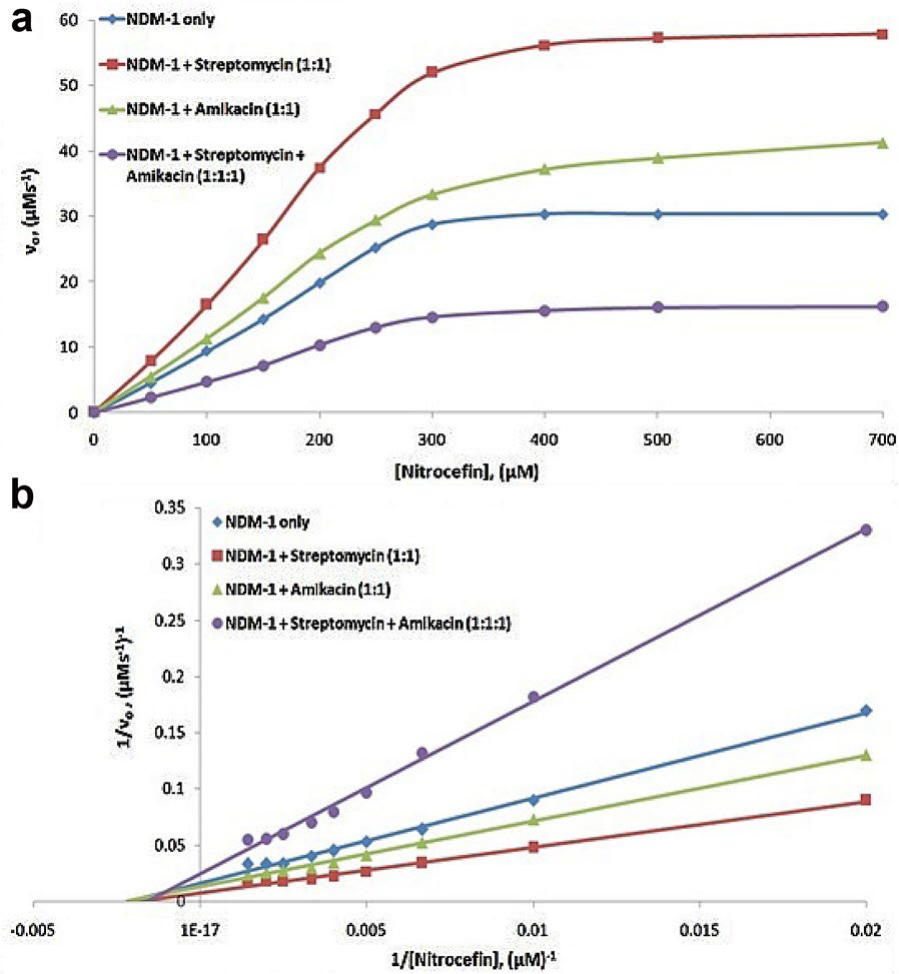
**a** Michaelis–Menten plot and **b** Lineweaver–Burk plot of nitrocefin hydrolysis by NDM-1 in the absence of any antibiotic, in the presence of 5 nM of streptomycin, in the presence of 5 nM of amikacin and in the presence of 5 nM each of streptomycin in combination with amikacin. 5 nM of NDM-1 was taken during the experiment. The study was done at 298 K using sodium phosphate buffer (50 mM) of pH 7.4

**Fig. 3:**
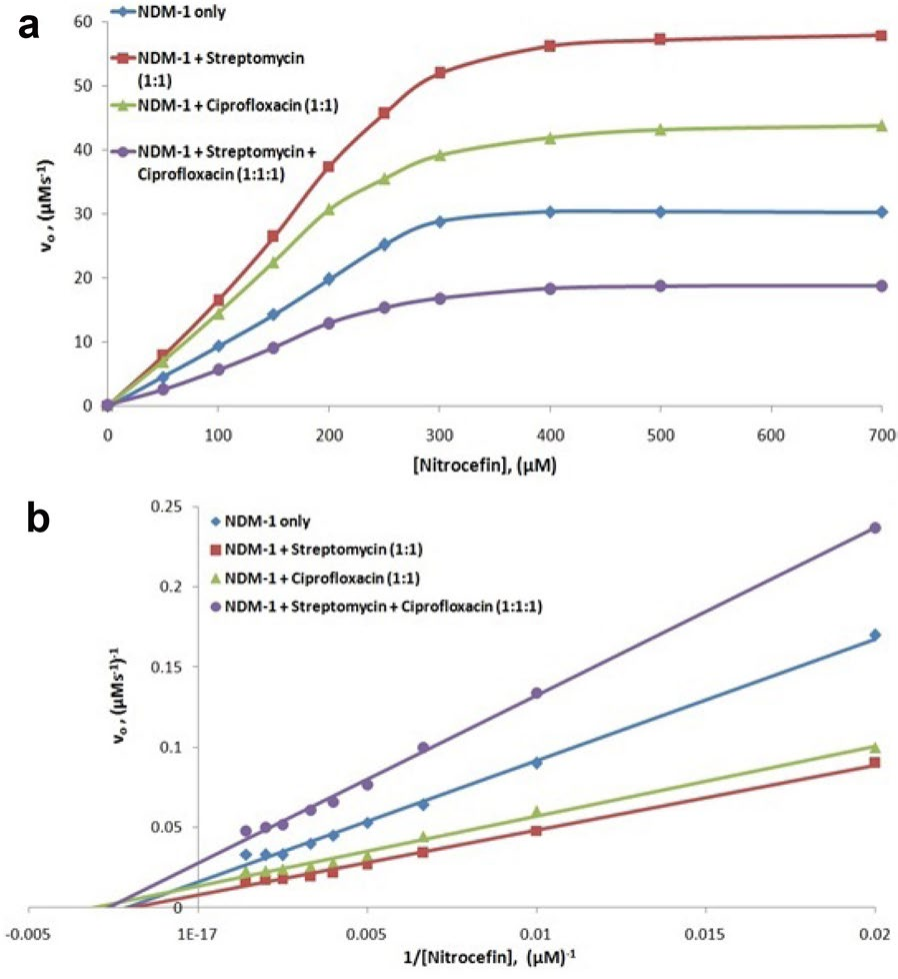
**a** Michaelis–Menten plot and **b** Lineweaver–Burk plot of nitrocefin hydrolysis by NDM-1 in the absence of any antibiotic, in the presence of 5 nM of streptomycin, in the presence of 5 nM of ciprofloxacin and in the presence of 5 nM each of streptomycin in combination with ciprofloxacin. 5 nM of NDM-1 was taken during the experiment. The study was done at 298 K using sodium phosphate buffer (50 mM) of pH 7.4

Enzyme kinetics study was performed to understand the effect of streptomycin and amikacin antibiotics binding in combination and streptomycin and ciprofloxacin antibiotics binding in combination on the catalytic efficiency and catalytic activity of NDM-1 β-lactamase enzyme. For the detection of NDM-1 β-lactamase activity upon binding of antibiotics in combination, nitrocefin was used as a substrate. From the obtained β-lactamase hydrolytic profile data, the Michaelis–Menten plots and the equivalent Lineweaver–Burk plots were plotted for the β-lactamase activity of the NDM-1 enzyme in the presence of streptomycin along with amikacin as shown in Fig. 2 and in the presence of streptomycin along with ciprofloxacin as shown in Fig. 3, respectively. Kinetic parameters such as Michaelis–Menten constant (*K*_m_), catalytic activity (*K*_cat_) and catalytic efficiency (*K*_cat_/*K*_m_) were calculated from the intercept and the slope of Lineweaver–Burk plot as shown in Table 3. The efficiency of nitrocefin hydrolysis by NDM-1 enzyme was increased from 27.337 to 48.816 µM^−1^s^−1^ in the presence of streptomycin and to 32.699 µM^−1^s^−1^ in the presence of amikacin. However, when streptomycin was taken in combination with amikacin then the efficiency of nitrocefin hydrolysis by NDM-1 enzyme was decreased to 13.838 µM^−1^s^−1^. The efficiency of nitrocefin hydrolysis by NDM-1 enzyme was increased from 27.337 to 48.816 µM^−1^s^−1^ in the presence of streptomycin and to 45.636 µM^−1^s^−1^ in the presence of ciprofloxacin. However, when streptomycin was taken in combination with ciprofloxacin, the efficiency of nitrocefin hydrolysis by NMD-1 enzyme was decreased to 19.230 µM^−1^s^−1^. The catalytic activity of nitrocefin hydrolysis by NDM-1 enzyme was decreased from 12,500 to 6666 s^−1^, respectively, when streptomycin was taken in combination with ciprofloxacin.

**Table 3:** Enzyme kinetic parameters of nitrocefin hydrolysis by the NDM-1 enzyme in the absence and presence of streptomycin and amikacin antibiotics in single and in combination as well as in the absence and presence of streptomycin and ciprofloxacin antibiotics in single and in combination

	*K*_m_ (µM)	*K*_cat_ (s^−1^)	*K*_cat_/*K*_m_ (µM^−1^s^−1^)
NDM-1	457.25	12,500	27.337
NDM-1 + streptomycin (1:1)	512.125	25,000	48.816
NDM-1 +amikacin (1:1)	509.66	16,666	32.699
NDM-1 +streptomycin + amikacin (1:1:1)	498.333	6896.4	13.838
NDM-1 +ciprofloxacin (1:1)	337.115	15,384.6	45.636
NDM-1 + streptomycin + ciprofloxacin (1:1:1)	346.632	6666	19.230

Data are reported as an average of ± standard error from three independent experiments

**Fig. 4:**
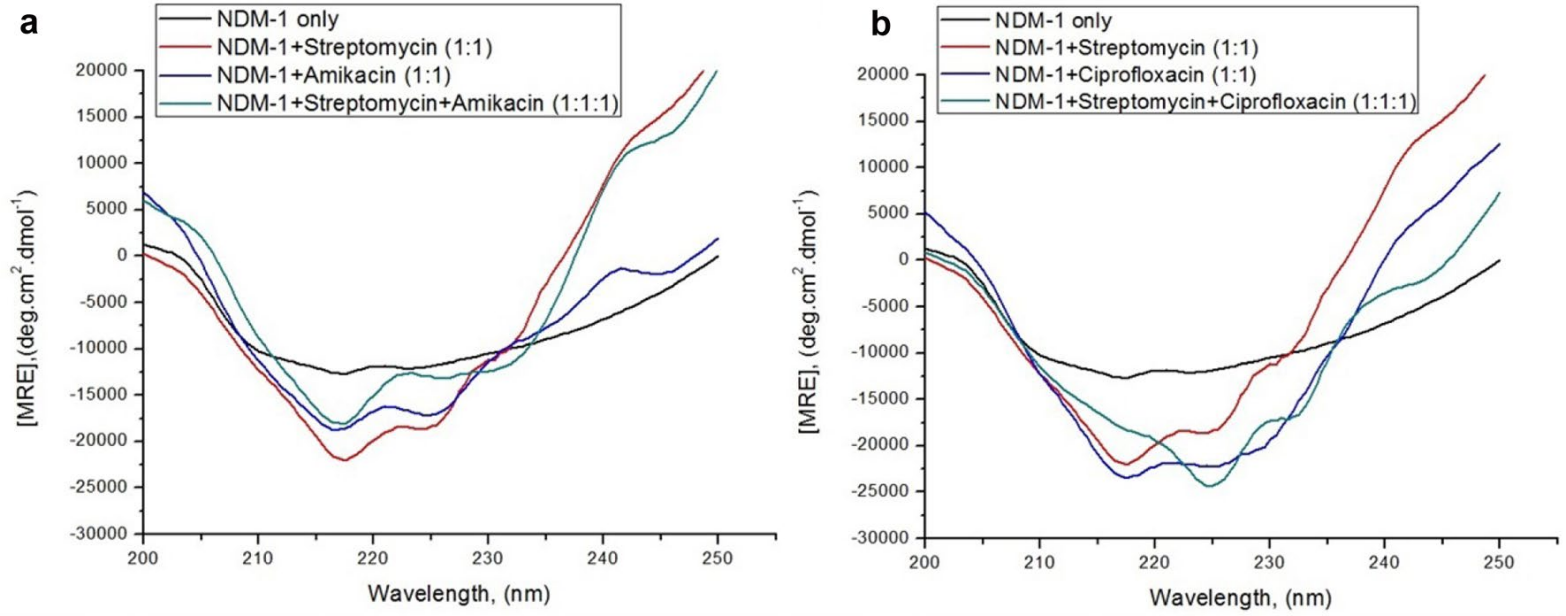
**a** CD spectral curves of NDM-1 (5 μM) taken alone, taken with streptomycin (5 μM), taken with amikacin (5 μM) and taken with streptomycin in combination with amikacin (5 μM each). **b** CD spectral curves of NDM-1 (5 μM) taken alone, taken with streptomycin (5 μM), taken with ciprofloxacin (5 μM) and taken with streptomycin in combination with ciprofloxacin (5 μM each). The experiments were carried out at 298 K in 50 mM sodium phosphate buffer of pH 7.4

Circular dichroism (CD) spectroscopy was performed to monitor the changes occurring in the conformation of the secondary structure of NDM-1, upon binding of single antibiotics and, in combinations. Four spectral curves were obtained while taking the CD spectra of NDM-1 in the absence and presence of single antibiotics in and, in combination. In the first case, the first spectra of NDM-1 protein were obtained in the absence of any antibiotic, the second one in the presence of streptomycin, the third one in the presence of amikacin and the fourth one in the presence of streptomycin taken along with amikacin as shown in panel A of Fig. 4. In the same way, in the second case, first CD spectra of NDM-1 protein were obtained in the absence of any antibiotic, second one in the presence of streptomycin, third one in the presence of ciprofloxacin and fourth one in the presence of streptomycin taken along with ciprofloxacin as shown in panel B of Fig. 4. Spectra were measured in far-UV range of 200–250 nm at 298 K. The MRE (mean residual ellipticity) values of NDM-1 protein at 218 nm and 222 nm and the percent α-helical content of NDM-1 protein at 222 nm in the absence and presence of antibiotics in single and in combination were calculated using Eqs. 5 and 6, respectively, as shown in Table 4. In the absence of any antibiotic, the MRE values of NDM-1 were found to be − 13153.0 and − 12, 591.0 deg cm^2^ dmol^−1^ at 218 and 222 nm, respectively, with the α-helical percentage of 33.83% at 222 nm. When streptomycin was taken in combination with amikacin, then the values of MRE of NDM-1 were changed to − 19,032.8 and − 13,515.9 deg cm^2^ dmol^−1^ at 218 and 222 nm, respectively, and the percentage of α-helical content was changed to 36.88% at 222 nm. When streptomycin was taken in combination with ciprofloxacin, then the values of MRE of NDM-1 were changed to − 18,885.6 and − 21,552.1 deg cm^2^ dmol^−1^ at 218 and 222 nm, respectively, and the percentage of α-helical content was changed to 63.40% at 222 nm.

**Table 4:** Spectral characteristic of NDM-1 protein in the absence of any antibiotic and in the presence of streptomycin and amikacin antibiotics in single and in combination as well as in the presence of streptomycin and ciprofloxacin antibiotics in single and in combination

	MRE_218_ (deg cm^2^ dmol^−1^)	MRE_222_ (deg cm^2^ dmol^−1^)	% α helix at 222
NDM-1 only	− 13,153.0 ± 110	− 12,591.0 ± 95	33.83
NDM-1 + streptomycin (1:1)	− 23,502.9 ± 103	− 18,963.4 ± 106	54.86
NDM-1 + amikacin (1:1)	− 19,347.2 ± 112	− 16,495.8 ± 126	46.71
NDM-1 + streptomycin + amikacin (1:1:1)	− 19,032.8 ± 131	− 13,515.9 ± 101	36.88
NDM-1 + ciprofloxacin (1:1)	− 24,311.2 ± 117	− 21,951.4 ± 98	64.72
NDM-1 + streptomycin + ciprofloxacin (1:1:1)	− 18,885.6 ± 124	− 21,552.1 ± 105	63.40

**Fig. 5:**
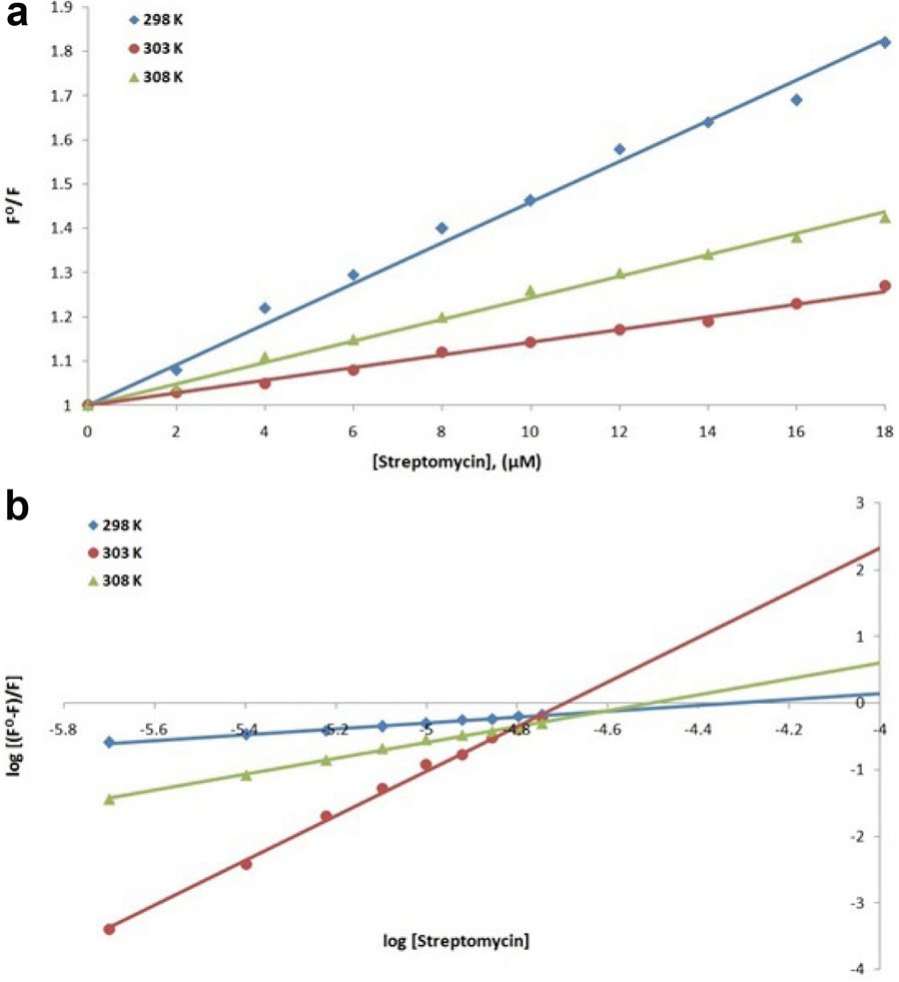
**a** Stern–Volmer plot of NDM-1 protein (2 μM) in the presence of streptomycin (0 to 18 μM) at 298, 303 and 308 K, in sodium phosphate buffer (50 mM) of pH 7.4. **b** Modified Stern–Volmer plot of NDM-1 protein (2 μM) in the presence of streptomycin (0 to 18 μM) at 298, 303 and 308 K, in sodium phosphate buffer (50 mM) of pH 7.4

**Fig. 6:**
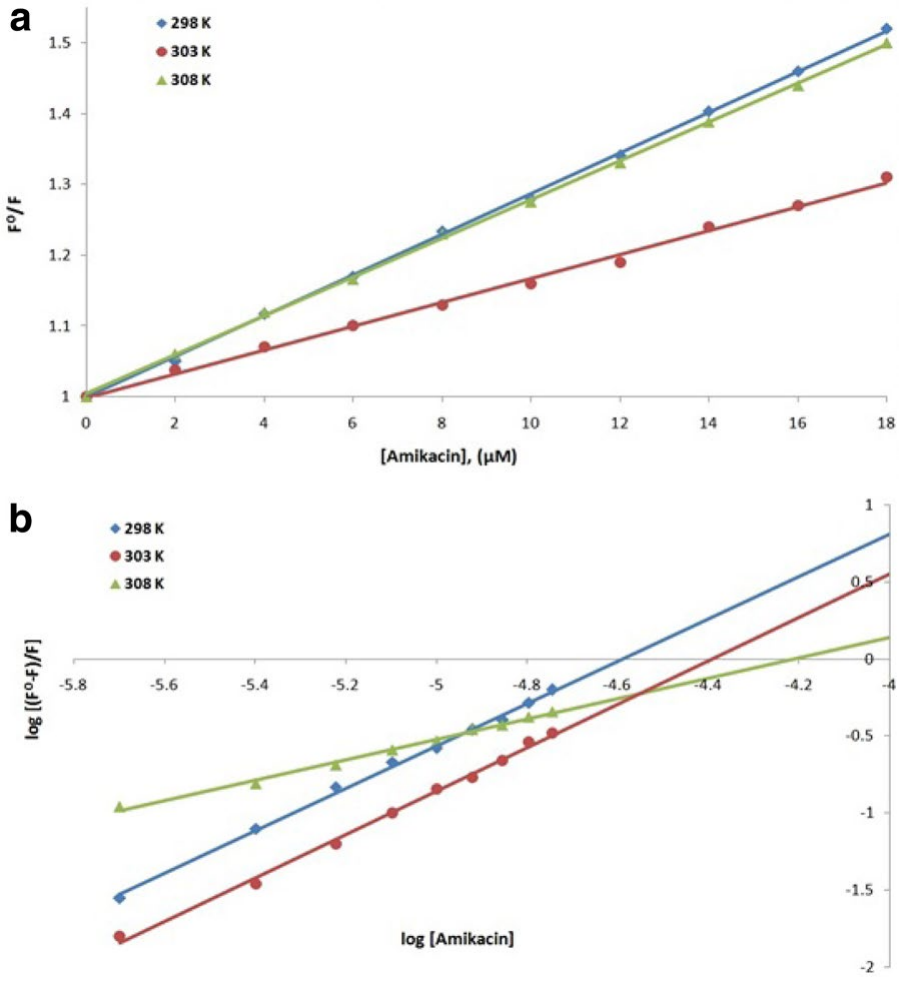
**a** Stern–Volmer plot of NDM-1 protein (2 μM) in the presence of amikacin (0 to 18 μM) at 298, 303 and 308 K, in sodium phosphate buffer (50 mM) of pH 7.4. **b** Modified Stern–Volmer plot of NDM-1 protein (2 μM) in the presence of amikacin (0 to 18 μM) at 298, 303 and 308 K, in sodium phosphate buffer (50 mM) of pH 7.4

**Fig. 7:**
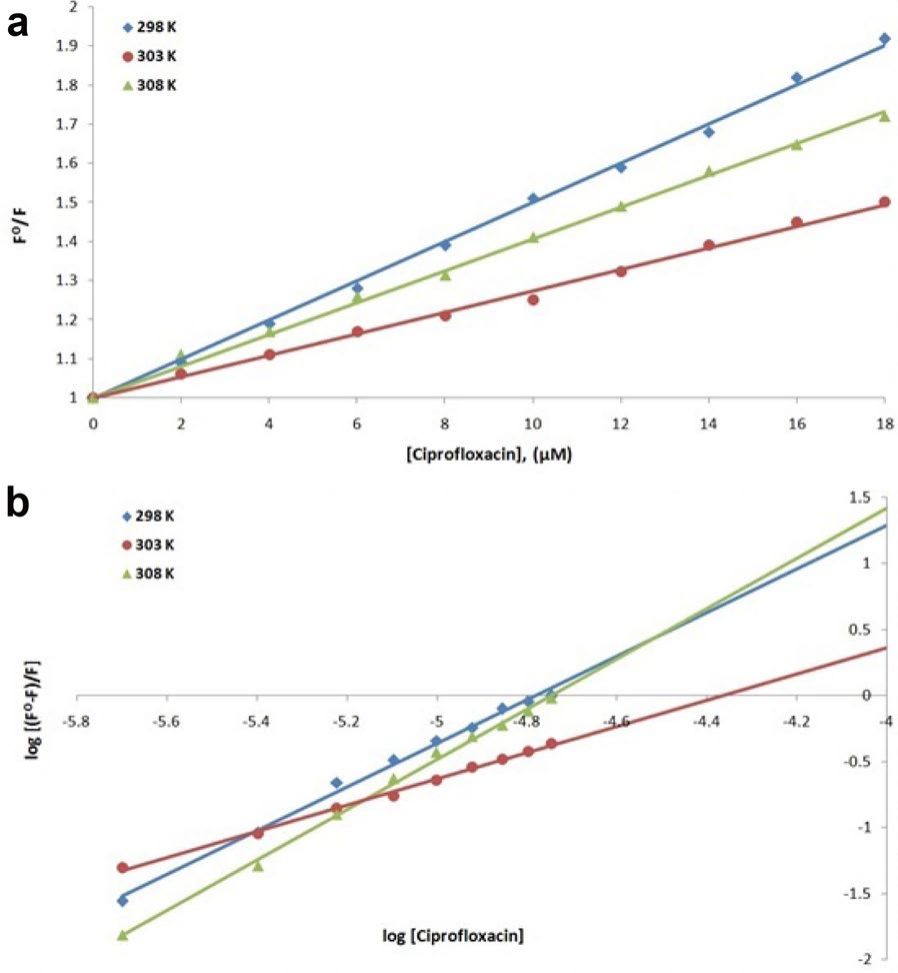
**a** Stern–Volmer plot of NDM-1 protein (2 μM) in the presence of ciprofloxacin (0 to 18 μM) at 298, 303 and 308 K, in sodium phosphate buffer (50 mM) of pH 7.4. **b** Modified Stern–Volmer plot of NDM-1 protein (2 μM) in the presence of ciprofloxacin (0 to 18 μM) at 298, 303 and 308 K, in sodium phosphate buffer (50 mM) of pH 7.4

**Fig. 8:**
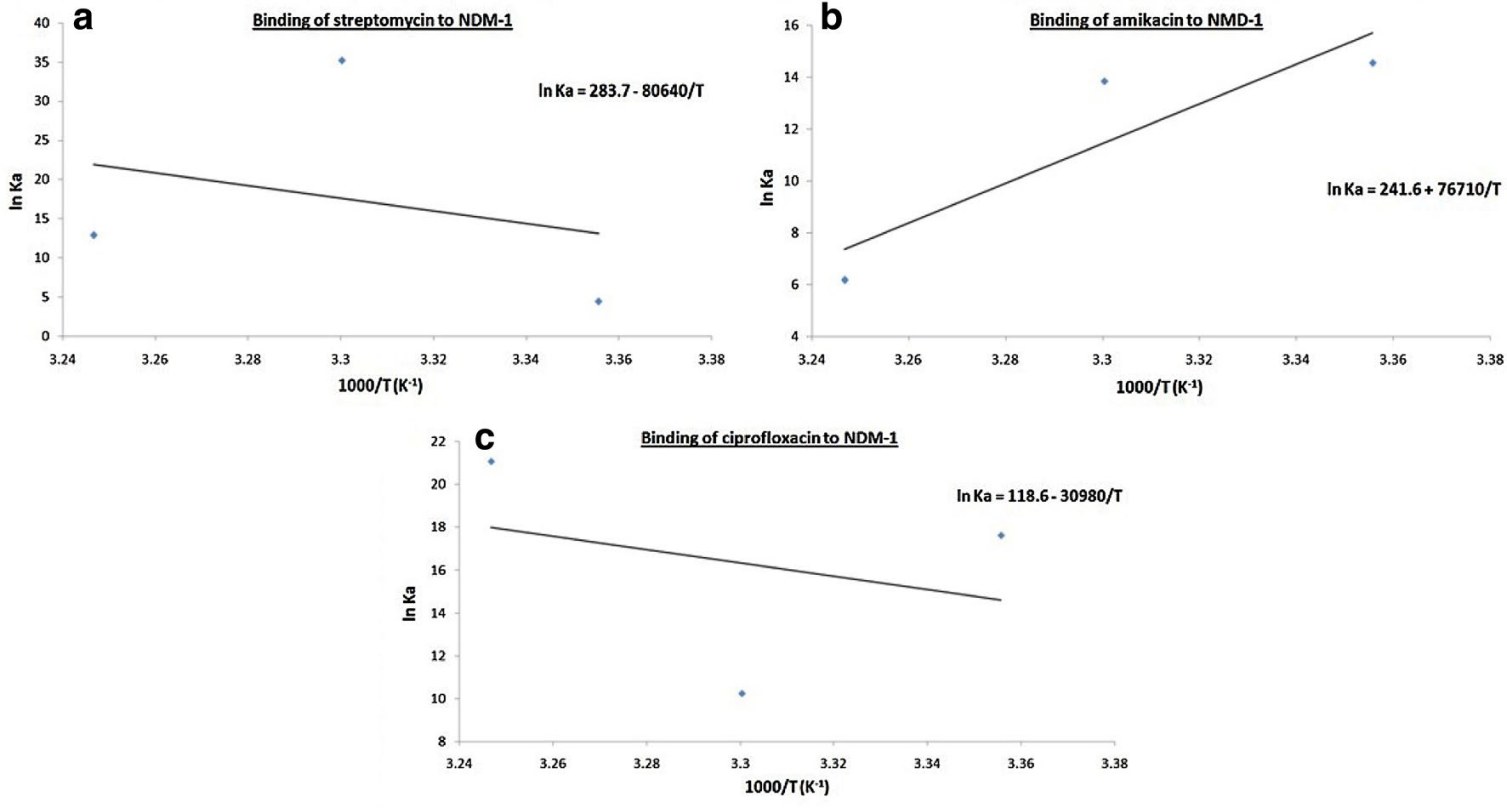
The figure shows Van’t Hoff plots for binding of streptomycin (**a**), amikacin (**b**) and ciprofloxacin (**c**) to NDM-1 (2 μM) at 298, 303 and 308 K. The concentration of streptomycin, amikacin and ciprofloxacin was varied from 0 to 18 μM in sodium phosphate buffer (50 mM) of pH 7.4

Fluorescence spectroscopy was performed to understand the interaction of a ligand with a protein. Therefore, to understand the interaction of streptomycin, amikacin and ciprofloxacin antibiotics with NDM-1 protein at 298, 303 and 308 K, fluorescence spectroscopic studies were carried out. Using the values at the emission maxima of the decrease in the fluorescence intensity curves, Stern–Volmer plot (panel A) and modified Stern–Volmer plot (panel B) were plotted of NDM-1 quenching by streptomycin (Fig. 5), amikacin (Fig. 6) and ciprofloxacin (Fig. 7). Using Stern–Volmer Eq. 7, *K*_sv_ (Stern–Volmer constant) was calculated and using modified Stern–Volmer Eq. 8, Ka (binding constant) and *n* (no. of antibiotic binding sites) were calculated as shown in Table 5. These constants are referred to as binding parameters. At all the three temperatures, the values of *K*_SV_ were found to be of the order of 10^4^ M^−1^ for each streptomycin, amikacin and ciprofloxacin binding. For NDM-1–streptomycin interaction the Ka values were calculated and were found in the range of 10^1^–10^15^ M^−1^, for NDM-1–amikacin interaction, the Ka values were calculated and were found in range of 10^2^–10^6^ M^−1^ whereas, for NDM-1–ciprofloxacin interaction, the Ka values were calculated as 10^4^–10^9^ M^−1^, respectively. Number of binding sites (*n*) accessible for binding of streptomycin was calculated to be 1 at 298 and 308 K and 3 at 303 K, for binding of amikacin it was calculated to be 1 at all the three temperatures and for binding of ciprofloxacin it was calculated to be 2 at 298 and 308 K and 1 at 303 K, respectively. Further, using the values of binding constants and temperatures, Van’t Hoff plot of streptomycin (Fig. 8a), amikacin (Fig. 8b) and ciprofloxacin (Fig. 8c) binding with NDM-1, were plotted. Using the slope of Van’t Hoff plots, the values of enthalpy change (ΔH) were calculated, and the values obtained were 670.440, − 637.766 and 257.657 Jmol^−1^ (Table 6), for NDM-1–streptomycin, NDM-1–amikacin and NDM-1–ciprofloxacin interaction, respectively. In the same way, using the intercept of Van’t Hoff plots, the values of entropy change (Δ*S*) were calculated, and the values obtained were 2385.681, − 2008.662 and 986.040 JK^−1^mol^−1^ (Table 6), for NDM-1–streptomycin, NDM-1–amikacin and NDM-1–ciprofloxacin interaction, respectively. Moreover, Gibbs free energy change (Δ*G*) were calculated using Van’t Hoff Eq. 9 and thermodynamic Eq. 10, and the obtained values were − 10.861, − 88.739 and − 32.979 kJmol^−1^ at 298, 303 and 308 K, respectively, for NDM-1–streptomycin interaction (Table 6). For NDM-1–amikacin interaction, the values were − 36.088, − 34.900 and − 15.797 kJmol^−1^ at 298, 303 and 308 K, respectively (Table 6). For NDM-1–ciprofloxacin interaction, the values were − 43.647, − 25.768 and − 54.015 kJmol^−1^ at 298, 303 and 308 K, respectively (Table 6).

**Table 5:** Binding parameters for interaction of NDM-1 with streptomycin, amikacin and ciprofloxacin

Antibiotic	Temperature (K)	*K*sv (M^−1^)	Ka (M^−1^)	*n*	*R*^2^
Streptomycin	298	4.4 × 10^4^	7.74 × 10^1^	0.435	0.992
	303	1.4 × 10^4^	1.99 × 10^15^	3.273	0.996
	308	2.4 × 10^4^	3.92 × 10^5^	1.243	0.996
Amikacin	298	2.8 × 10^4^	2.12 × 10^6^	1.379	0.993
	303	1.6 × 10^4^	1.04 × 10^6^	1.382	0.995
	308	2.8 × 10^4^	4.78 × 10^2^	0.639	0.994
Ciprofloxacin	298	4.8 × 10^4^	4.48 × 10^7^	1.604	0.994
	303	2.8 × 10^4^	2.77 × 10^4^	1.012	0.996
	308	4.0 × 10^4^	1.45 × 10^9^	1.930	0.997

**Table 6:** Thermodynamic parameters for interaction of NDM-1 with streptomycin, amikacin and ciprofloxacin

Antibiotic	Temperature (K)	Δ*H* (Jmol^−1^)	Δ*S* (J K^−1^mol^−1^)	*T*Δ*S* (kJ mol^−1^)	Δ*G* (kJ mol^−1^)
Streptomycin	298	670.440 ± 0.5	2385.681 ± 0.8	710.932 ± 0.7	− 10.861 ± 1.2
	303			722.861 ± 1.0	− 88.739 ± 1.7
	308			734.789 ± 1.3	− 32.979 ± 1.4
Amikacin	298	− 637.766 ± 0.6	− 2008.662 ± 1.2	− 598.581 ± 0.3	− 36.088 ± 1.5
	303			− 608.624 ± 0.8	− 34.900 ± 1.1
	308			− 618.667 ± 1.6	− 15.797 ± 0.9
Ciprofloxacin	298	257.657 ± 0.7	986.040 ± 1.4	293.839 ± 0.8	− 43.647 ± 0.9
	303			298.770 ± 1.1	− 25.768 ± 1.5
	308			303.700 ± 1.8	− 54.015 ± 1.3

## Discussion

The AK-66 strain of *Klebsiella pneumoniae* having NDM-1 gene on its plasmid showed resistance towards an aminoglycoside antibiotic streptomycin with the MIC of 1024 µg/ml, another aminoglycoside antibiotic amikacin with MIC greater than 1024 µg/ml and a quinolone antibiotic ciprofloxacin with the MIC of 128 µg/ml.

When two antibiotics work together to produce a potential effect in the treatment of an infection, compared to its use as a single antibiotic, is referred to as synergy. Synergy is observed when the fractional inhibitory concentration index (FICI) value calculated is less than or equal to 0.5 [26, 34]. In this study, the FICI value of streptomycin and amikacin combination was observed to be 0.3125 and the FICI value of streptomycin and ciprofloxacin combination was 0.31 for NDM-1 gene carrying the AK-66 strain of *Klebsiella pneumoniae.* Since both the FICI values are less than 0.5, streptomycin and amikacin combination and streptomycin and ciprofloxacin combination showed synergistic effect towards NDM-1 producing bacterial strain.

Since streptomycin and amikacin combination and streptomycin and ciprofloxacin combination show synergistic effect towards NDM-1 producing bacterial strains, binding of these antibiotic combinations might induce some inhibitory effect on NDM-1 β-lactamase. Hence, enzyme kinetics was performed to monitor the combination therapy of two aminoglycosides and an aminoglycoside with quinolone on the catalytic activity and catalytic efficiency of NDM-1 producing bacteria. The rate of nitrocefin appearance and disappearance was observed as a measure of NDM-1 β-lactamase activity since nitrocefin is used to detect the presence of β-lactamase and gives a characteristic β-lactamase hydrolytic profile [35]. When NDM-1 enzyme was treated with streptomycin along with amikacin in 1 times molar ratio of each with respect to the NDM-1 enzyme, then the catalytic efficiency of the NDM-1 enzyme to hydrolyze nitrocefin was decreased by about 49.37% and the catalytic activity of the enzyme was decreased by 44.82%. In the same way, when NDM-1 enzyme was treated with streptomycin along with ciprofloxacin in 1 times molar ratio of each, with respect to the NDM-1 enzyme, then the catalytic efficiency of the NDM-1 enzyme to hydrolyze nitrocefin was decreased by about 29.65% and the catalytic activity of enzyme was decreased by 46.67%. This decrease in the catalytic activity and catalytic efficiency of the NDM-1 enzyme is probably due to some modifications occurring in the overall structure of enzyme or changes induced in the residues present near the active site. Therefore, 5 nM each of streptomycin and amikacin combination and 5 nM each of streptomycin and ciprofloxacin combination have the ability to inhibit the catalytic efficiency and catalytic activity of the NDM-1 enzyme.

The secondary structure of any protein has various conformations such as α-helix, β-sheet, random coil, beta-turn, etc. CD spectroscopic studies yield valuable information about the secondary state conformations of the protein molecules mainly the fraction of protein molecules present in the form of α-helices [32]. It was observed from Fig. 4 that NDM-1 protein in the absence of any antibiotics showed two peaks at 218 and 222 nm with 33.83% α-helical content at 222 nm. This curve was quite comparable to the reported CD curve of the NDM-1 protein having 30–34% α-helical content, respectively [24]. In the presence of streptomycin antibiotic, the α-helical content of NDM-1 was increased by 62.16% at 222 nm. In the presence of amikacin antibiotic, 91.30% increase and in the presence of ciprofloxacin antibiotic, 38.07% increase in the α-helical content of NDM-1 protein was observed. In the presence of streptomycin in combination with amikacin, the α-helical content of NMD-1 protein was increased by about 87.40%, inferring that upon binding of these two antibiotics in combination quite an abrupt change in the conformation of NDM-1 which enhanced α-helical content from 33.83 to 63.40%. However, in the presence of streptomycin in combination with ciprofloxacin, the α-helical content of NMD-1 protein was increased by about 9.015%, implying that upon binding of these two antibiotics in combination the α-helical content of NDM-1 underwent changes from 33.83% to 54.86% and 46.71% to finally 36.88%, bringing several modifications in the structural assembly of NDM-1 protein. Hence, substantial disruption in the secondary structure of the NDM-1 protein was observed upon binding of streptomycin and amikacin in combination, and streptomycin and ciprofloxacin in combination.

Fluorescence spectroscopy helps us to get information about the interaction of drugs with a particular protein, the mechanism behind its action and the effect it produces on the protein. Fluorescence is observed due to absorption of energy in between electronic energy levels and its further emission at a longer wavelength with loss of some energy in the vibrational form to the surrounding [36, 37]. Upon binding of the drug with a protein, quenching (a progressive decrease in the fluorescence intensity curves) is observed which is recorded during the fluorescence spectroscopy [38, 39]. Complex formation, energy transfers, molecular collision and reactions in the excited state are responsible for quenching phenomenon [40]. Quenching is either dynamic or static depending on whether quenching occurred due to excited state collisions or due to the formation of ground-state complexes [41]. Hence in this study to understand the interaction of streptomycin, amikacin and ciprofloxacin antibiotics with NDM-1 protein, fluorescence spectroscopy was performed. At 298, 303 and 308 K, linear quenching of NDM-1 fluorescence was observed upon rise in the concentration of antibiotics as seen from Stern–Volmer plots signifying that upon binding of antibiotics, structural changes occur in the NDM-1 protein and it also indicates that aromatic amino acid residues such as tryptophan, etc., are present in the close proximity of antibiotic binding site. There is a significant interaction between streptomycin and NDM-1 protein, amikacin and NDM-1 protein and ciprofloxacin and NDM-1 protein as observed from the *K*sv values of the order of 10^4^ M^−1^ in all the three antibiotic binding cases. Ka for streptomycin–NDM-1 interaction was found to be of the order of 10^1^–10^15^ M^−1^, which indicated that there is medium to very strong interaction between the two, for amikacin–NDM-1 interaction, Ka of the order of 10^2^–10^6^ M^−1^ indicated that there is strong interaction between the two and for ciprofloxacin–NDM-1 interaction, Ka of the order of 10^4^–10^9^ M^−1^ indicates that there is strong to very strong interaction between the two. Moreover, it was observed that at 298 K, streptomycin and amikacin have a single binding site on NDM-1 protein and ciprofloxacin have two binding sites on the NDM-1 protein. After monitoring thermodynamic parameters it was observed that the values of ΔH (change in enthalpy) for streptomycin–NDM-1 interaction and ciprofloxacin–NDM-1 interaction were positive signifying endothermic reaction undergoing in their interaction process. In the same way, the value of ΔH for amikacin–NDM-1 interaction was negative signifying exothermic process undergoing in their interaction process. Increase in the disorderness of the system was observed upon the interaction of streptomycin and ciprofloxacin with NDM-1 protein because there ΔS is positive, whereas, the decrease in the disorderness of the system was observed upon the interaction of amikacin with NMD-1 protein because of their ΔS negative value. At all the three temperatures and in all antibiotic binding cases, the values of ΔG were negative indicating that the binding of streptomycin, amikacin and ciprofloxacin with NDM-1 was a spontaneous process.

The results show similar synergistic effect of antibiotic combinations towards resistant beta-lactamase carrying bacterial strains, as shown in our previous studies, where cefotaxime–streptomycin combination, cefotaxime–doripenem combination and cefotaxime–aztreonam combination were proposed, working effectively against CTX-M-15 beta-lactamase producing bacterial strains [15–17]. Another study was carried out by our group similar to present study in which we proposed two effective combinations of antibiotics, doripenem–cefoxitin and doripenem–tetracycline working against NDM-1 beta-lactamase producing bacterial strains [18].

It is essential to mention that in 2016 FDA updated a warning about the association of fluoroquinolones with disabling and potentially permanent side-effects. So the fluoroquinolones should be used after weighing the risks and benefits in cases of high antimicrobial resistance and no available options [42].

## Conclusions

The study concludes that the combination of streptomycin–amikacin and streptomycin–ciprofloxacin shows synergistic effect towards bacterial strains producing NDM-1. The efficiency of NDM-1 enzyme is substantially reduced in the presence of streptomycin and amikacin combination and streptomycin and ciprofloxacin combination. Hence, the study proposes the combination of either two aminoglycoside or an aminoglycoside and a quinolone as a possible therapeutic option against multi-drug-resistant NDM-1 producing bacterial strains.

## Data Availability

All data generated or analyzed during this study are included in this published article (and its Additional files).
